# Carers’ experiences of involvement in care planning: a qualitative exploration of the facilitators and barriers to engagement with mental health services

**DOI:** 10.1186/s12888-015-0590-y

**Published:** 2015-08-29

**Authors:** Lindsey Cree, Helen L. Brooks, Kathryn Berzins, Claire Fraser, Karina Lovell, Penny Bee

**Affiliations:** EQUIP, School of Nursing, Midwifery and Social Work, Jean McFarlane Building, University of Manchester, Oxford Road, Manchester, M13 9PL UK; School of Healthcare, University of Leeds, ᅟ, Leeds, LS2 9JT UK; Manchester Mental Health and Social Care Trust, University of Leeds, ᅟ, Manchester, ᅟ UK

## Abstract

**Background:**

Formal recognition and involvement of carers in mental health services has been the focus of recent policy and practice initiatives as well as being supported by carers themselves. However, carers still report feeling marginalised and distanced from services. A prominent theme is that that they are not listened to and their concerns are not taken seriously. Compared to service user views, the reasons underpinning carers’ dissatisfaction with care-planning procedures have been relatively neglected in the research literature, despite the substantial and significant contribution that they make to mental health services. The aim of the study was to explore carers’ experiences of the care planning process for people with severe mental illness.

**Methods:**

Qualitative interviews and focus groups were undertaken with carers. Data were combined and analysed using framework analysis.

**Results:**

Whilst identifying a shared desire for involvement and confirming a potential role for carers within services, our data highlighted that many carers perceive a lack of involvement in care planning and a lack of recognition and appreciation of their role from health professionals. Barriers to involvement included structural barriers, such as the timing and location of meetings, cultural barriers relating to power imbalances within the system and specific barriers relating to confidentiality.

**Conclusions:**

This qualitative study led by a researcher who was a carer herself has developed the understanding of the potential role of carers within the care planning process within mental health services, along with the facilitators and barriers to achieving optimal involvement.

## Background

The prevalence of mental health problems in the UK is such that at any given time, one in four adults will experience at least one or more mental disorders [1]. Globally, mental health problems are estimated to affect 450 million people [2]. Many people who experience severe mental health problems are supported by a carer, defined as “anyone who cares, unpaid, for a friend or family member who due to illness, disability, a mental health problem or an addiction cannot cope without their support” [3]. The number of individuals caring for somebody with a mental health difficulty in the UK is estimated to be 1.5 million [4]. Together, these individuals represent a significant source of resources to health services, and are posited to save statutory services between £119 million and £1.24 billion in nursing care costs each year [5, 6]. Effectively supporting carers is thus crucial to the economy and delivery of cost-effective health care.

Undeniably, carers have the potential to contribute a huge amount of expertise, experience and knowledge to the services, and consultations in which they become involved, across both voluntary and statutory settings [7]. Their influence can be seen at both service development level and at individual patient level where they can provide information crucial to risk assessments and care planning and ensure the instigation of care that is in the best interests of both service user and carer. At a relational level, service user/carer involvement has been advocated to improve the relationship between patient and practitioner [8].

Formal recognition of the role and value of carers has been increased through a series of legislative measures initiated over the past 15 years [9, 10]. The application of the new Mental Health Acts in England, Wales and Scotland were identified as valuable opportunities for services to make real changes to care planning involvement, and to ensure that wherever possible carers are involved in developing service users’ care plans [11]. These initiatives are additionally supported by other government policies, including the National Carers Strategy [12–15].

Professional guidance and good practice documents have been issued that provide advice to mental health professionals on promoting carer involvement [16–18]. As a result, the concept of carer involvement in mental health care planning process has become embedded within policy and service ideologies, and ise strongly supported by carers themselves [19, 20].

### The experience of being a carer

It is well documented that service engagement with family members in treatment programmes can improve health outcomes for service users [21, 22]. In line with this, current best practice guidance states that the outcome of any clinical assessment will be a care plan developed collaboratively between the service user, health professionals and family members and carers where appropriate [23, 24]. Yet despite this apparently supportive broader context, research on carers’ experiences of involvement within mental health services is scant, particularly in comparison to the body of research published on service user involvement. The research conducted has tended to focus on the difficulties and negative effects of providing care or the ‘caregiver burden’ [25–27], the negative impact of dysfunctional family environments [28], has combined carer and service user data or been purely survey based. Fewer studies have looked in depth at carers’ experiences in their own right. The limited data that are available have reported high levels of carer dissatisfaction with involvement in both inpatient and community settings [29] and that carers would like to obtain more information on services and be more involved in care consultations [30, 31]. A recent review found that both service users and carers wanted increased involvement, specifically in the assessment and planning of care [32]. Other data suggest that carers often feel ignored or neglected by mental health services [33–35], the common perception being that they are not listened to and their concerns are not taken seriously [36].

### Barriers to involving carers in health services

One frequently cited barrier to carer involvement in care planning is the right of the service user to confidentiality, which can prevent information being shared with carers by services. From a health professional perspective, some of the difficulties  about confidentiality and information sharing are ethical and legal obligations, with a breach of confidence potentially resulting in disciplinary measures and legal proceedings. Barriers erected because of perceived rather than actual confidentiality issues can limit participation and dialogue between carers, the people that they care for, and professionals [37]. It is believed that professionals are not always confident in where the limits of confidentiality lie, and that further training relating to specific legal requirements may be beneficial [38].

This study aims to address the aforementioned gap in existing literature by examining, in depth, carers' experiences of mental health services and care planning in particular. This article therefore reports qualitative data describing carers' experiences with involvement in care planning, exploring both barriers and potential facilitators. This study was part of the wider Enhancing the quality of user involved care planning in Mental Health Services (EQUIP) project, designed to improve user and carer involvement in mental health services, and was led by a carer researcher.

### Aim

To explore carers’ experiences of the care planning process for people with severe mental illness.

## Methods

### Study design

The study utilised a qualitative design incorporating both focus groups and interviews with people who cared for someone with a severe mental illness, to explore their views on user/carer involved care planning. Themes identified in the focus groups (Study 1) were explored in more depth with participants during the in-depth one-to-one interviews (Study 2). Study 1 was completed prior to Study 2 commencement. The use of focus groups as a sole method of data collection has been discouraged due to the potential of missing important issues that participants might not raise in front of others [39]. Therefore semi-structured interviews and focus groups were used in conjunction with each other to explore the experience of caring for someone with a severe mental illness. Participants took part in either a focus group or a one-to-one interview.

Ethical approval was granted by the NRES Committee North West - Greater Manchester North and Dyfed Powys Research Ethics Committee (ref: 13/NW/0047 and ref: 13/WA/0074).

### Participants

The target population was carers of users of secondary care mental health services, recruited from one of two mental health trusts in the UK. Inclusion criteria for this study were that participants were caring for relative with serious mental illness, aged 18 or over, and had capacity to consent. Participants were recruited using a range of methods including advertising the study on trust intranets, Twitter, newsletters and press releases. In addition, posters were displayed and flyers distributed in a wide range of locations (hospital sites, assertive outreach teams, community mental health teams, community centres, carer and service user groups). Participants were sampled purposively to ensure a diversity of opinion in relation to the topic under consideration.

Carers who were interested in participating in the study contacted the research team who arranged a convenient date for an interview or focus group. Carers received a detailed information sheet about the study at least 24 hours before the interview/focus group took place. The procedure was explained again immediately prior to the research being undertaken and participants were given the opportunity to ask any questions prior to written, informed consent being obtained.

### Data collection

The purpose of the focus groups and interviews was to gather information about experiences of being a carer, their involvement in mental health care planning, and how, if necessary, this could be improved. Focus groups also explored what kind of training staff might need to enhance engagement with service users and carers, and to improve the care planning process. The interview and focus group schedules were developed with input from service users and carers on the advisory group for the project, and were informed by a recent review conducted in relation to care planning [32].

Four focus groups were carried out with service users and carers, which included 14 carers, three of whom also considered themselves to be service users. An additional focus group was carried out with service users, carers and professionals (3 carers, see Table 1, service user and professional data have been presented separately elsewhere). Once the focus groups were complete, semi-structured interview schedules were adapted in line with data collected from the focus groups, and then 26 one-to-one semi-structured interviews were also carried out with carers.

**Table 1:** Demographic information relating to participants

	Total	Gender		Ethnicity	
		Male	Female	White	Black and Minority Ethnic (BME)
Focus groups					
Carer	11	1	10	8	3
Service user and carer	3	1	2	3	0
Interviews					
Carer	26	6	20	22	4

Focus groups and semi-structured interviews lasted approximately 60–90 minutes and were undertaken at a range of locations to support participation: at university campuses, trust sites and community locations, e.g. at carers' centres and participant's homes. Participants received high street gift vouchers worth £25 for taking part. Focus groups were carried out by a team of two or three researchers who covered the roles of lead facilitator, co-facilitator/scribe and meeting/greeting/governance and welfare. The research team who undertook the focus groups included a user/carer researcher where possible.

Carers (*n* = 37) were given debriefing sheets at the end of each focus group or interview. A distress protocol was in place should any participant have shown signs of distress; however, this was not required during any focus group or interview. All focus groups and interviews were digitally audio-recorded, transcribed verbatim and anonymised prior to data analysis.

### Data analysis

Anonymised transcripts from the semi-structured interviews and focus groups were analysed using Framework Analysis. Framework Analysis is commonly used within qualitative health research and allows for both inductive and deductive coding to be incorporated into the analysis process, which means that codes emerging from the data can be combined with important codes that were identified prior to the study [40].

The analysis team comprised service user and carer co-applicants working alongside experienced qualitative researchers for independent coding purposes. Authors read their transcripts on multiple occasions to familiarise themselves with the data before starting to code the transcripts. The data and analysis were managed using an excel database comprising participant characteristics, along with a word document containing emerging themes from each transcript, to provide a data trail. Authors met regularly to discuss their own emergent codes, to develop a provisional coding framework, to discuss alternative explanations of interpretations and to ensure that the emerging codes remained grounded in the original data for purposes of validity. This approach to analysis meant that during the constant comparison of new data, the provisional framework was amended and re-shaped to enable the introduction of new codes, and allowed for the removal of other codes that became superfluous over the course of the analysis. The resultant framework contained only those codes agreed upon by the whole analysis team. Previous iterations of the coding framework were stored for purposes of transparency and the research team agreed as a whole when data saturation had occurred and no new themes were emerging from the data.

In order to further strengthen the validity of the qualitative findings, the final coding framework was presented to the wider study team, who was asked to comment on whether the framework seemed grounded in the data, on any ommissions in the framework or any ambiguities.

## Results

The results from the focus groups and interviews are combined and presented under key themes, which are each illustrated by quotations taken directly from the data chosen to provide rich detail and contextual information. The themes include carers’ current experiences within health services, as well as care planning specifically. Data are provided on optimal care planning, and barriers and facilitators to obtaining this optimal experience from the perspectives of carers.

### The structure and purpose of optimal care planning

There was a shared desire for care planning amongst participants at least in principal. Carers had well defined ideas about what a good care plan should look like, and what it should be used for. Participants felt that a good care plan would have shared aims and would involve input from all relevant parties (service user, professionals and carers). It should be written down and structured in such a way as to contain information about anticipated timescales demonstrating progress, but also accommodating setbacks and change.

*“What good care planning would be like? For us all to sit down and to build a picture of what my son would like to be doing in six months’ time and how he would like to get there. And for us all to have a written copy of how that’s going to happen and somebody to follow it through every stage of the way.”* Carer1001

A good care plan should be concise and incorporate positive outcomes for the carers and families as well as for the service user. There was a general consensus that much of the information provided by services was unnecessary and providing information in a more relevant and condensed way would facilitate engagement with service users or carers.

*“We only need one agreement of where we’re headed, that’s all. Not a file full of information that hardly anyone looks at. Yeah, that would be a good result for me. If there was just one sheet of paper, with aims and objective, and…you know what I mean?”* Carer 1002

One of the most important purposes of a care plan for carerswas the provision of a statement of the services which would be provided. In this sense, care plans were perceived to have a potentially important role to play in service accountability. In line with this, the formal recording of timescales for actions was perceived to be important for carers and gave them something to refer back to during the course of their contact with services.

*“It kind of regulates people …when you’ve got something written, it’s very different than just by word of mouth, sort of thing, it’s… contractual, you know who is responsible for what…the needs are identified and you can see how and when those needs are met, the improvement that can bring to a person’s life really …something you can rely on as well, you’re not just left in the wind, well, who is going to do what, what should I do, what am I, you know, you’re identifying who can do what and when and you feel it’s like a support…a network, it’s like a support mechanism.”* Carer 1003

There were two secondary purposes of the care plan: as an aid to recovery and a means of improving communication between everybody involved in the service user’s care. However, the care plan had to be regularly reviewed and adapted, as carers were sensitive to service users being made to feel they had failed to achieve goals. Flexibility of the care plan should also allow for changes in people’s wishes and variations in their health.

*“all people involved professionally and whatever, in the person that the care plan is for, all to be together and discuss openly, what part they play in the needs and journey, and recovery of the individual … a tool that’s put in place, to work towards somebody’s recovery … for putting the right, correct care in place for the individual.”* Carer1004

### The potential versus the actual role of carers within mental health care planning

Participants talked about and acknowledged the unique and valuable role that carers could play in mental health services and care planning in particular. This related to the deep knowledge they had of the service user, which might otherwise be inaccessible to services. Thid knowledge, gained over the length of the time they had known the service user, enabled carers to develop a more detailed and holistic understanding, having known the person both when they were well and unwell. Additionally, carers could contribute knowledge about their interests and motivations, which the service user may feel unable to impart.

*“The first thing is some knowledge of the individual, who they are.. what are the kinds of things that will motivate them, what will demotivate them?”* Carer1005

The perceived carer expertise appeared to lie in seeing the whole person, including individual longitudinal trajectories as well as their other needs (including physical and social in addition to mental health needs). One of the major concerns carers had about current care planning, was that service users’ physical health needs were not being met alongside their mental illness. In some cases, they felt that physical illnesses were entirely omitted from current care plans, and this reflected a similar problem in wider health records, whereby mental illness was often not recorded on physical health records. This was often attributed to a lack of connection between mental and physical health services, and became particularly problematic when service users were hospitalised in a crisis, for either their mental or physical health problem.

*“and me and my son were ringing and ringing, we found that the GP…. knew about his strokes. But the CPN, the psychiatrist had nothing on their records about his strokes.”* Carer 1006

*“(psychiatrist) had no idea, or aware of how my sister sits there, she has an incontinence problem”.* Carer 1007

One frequently mentioned aspect of carers’ knowledge was the ability to identify early warning signs of relapse, which may become useful for crisis planning elements of the care plan.

*“ I can tell, I’ve got like a little pointer thing, when Michael is starting to go off and… when his, when his voice changes, that’s the first sign”.* Carer 1008

Participants felt that this unique knowledge became even more important when it was seen in the wider context of the consultation, where relationships between the service user and health professional may be strained. For example, carers cited being able to provide objectivity in examples of situations where service users were not always honest with health professionals for a myriad of reasons. Whilst all contributions to care planning were considered valuable, combining input from all three parties (service user, carer and professional) could lead to a final care plan that was greater that the whole of its individual parts. The role of carer, in particular,  became heightened given the current economic climate, which limited both time and human resources, and often made it was difficult for professionals to fully get to know service users.

*“…the care plan should be drawn up in conjunction with… the service user, plus also the carer who can provide objective input at the time to say well she might agree to do that but, in fact, she won’t do that so it’s waste of time.”* Carer 1007

*“because everybody has got timetables and…and places to be at certain times and so many different other…so many different…so many clients, probably, to see, in a short space of time.”* Carer 1002

Despite the potential value attributed to involving carers in care planning, current experiences of care planning within mental health services amongst the majority of our participants failed to meet that potential. This appeared to be related to a negative perception of care planning generally within services.

### Current experiences of care planning within mental health services

Consensus amongst the carers who were included in our study was that their experiences of care planning were generally negative. Several carers said that there was no care plan in place at all, despite carers and service users directly requesting them from health professionals, and others were unsure care plans existed because they had not seen one.

*“not once was a care plan ever mentioned for Helen, not in all the time we went to XXX Hospital and that was, say, what, about eight years, nine years…? And not one person ever mentioned a care plan to us. We did mention it and they turned around and said… oh, erm, we’ve no need to get onto that yet, we’ll get onto that later.”* Carer 1009

Of those who did have direct experience of the care planning process, the experiences remained largely negative. Specifically, this related to completed care plans being described as tokenistic and meaningless. Some carers commented that care plans often became lost within other administrative paperwork, were not reviewed sufficiently, and, as a result, very few people referred to them. The strength of these opinions was significant and demonstrated a perception that care planning was used by services as a means of perpetuating top down, risk averse cultures, thereby reaffirming boundaries between patient and service user roles within services.

*“I think it's lip service, absolute lip service. It's just… a piece of paper that somebody writes things down on, just in case they're questioned at some later stage, to protect… them, not actually…because a lot of it wasn't carried out… for somebody to just say, oh, you know, oh good, we've seen him and that's it … Right, tick it in the box. It's box ticking.”* Carer 1010

Simultaneously, carers demonstrated cynicism about whether what was actually documented in care plans would be enacted in practice, or would have any bearing on the experience of service users and carers. This cynicism seemed grounded in direct experience as participants reported initially being enthusiastic about the process of care planning, but this generally eroded over time as they experienced first-hand how it was regarded, managed, and used within services.

*“It didn't appear that the notes that were made at the review were actually implemented by all the people involved in his care. So…the goalposts seemed to be constantly moving, which made it difficult for us then, to have a consistent approach that helped us all in his care … we did often find that we were in challenging situations where, at a review previous, they'd said something and then at another review, they were saying something completely different. And you would then draw their attention to what was said previously and it made it very uncomfortable.”* Carer 1010

### Facilitators to involving carers in the process of care planning

The majority of carers talked about the need for improved consultation between the service user, carers and professionals. This consultation process for carers could be hampered by their lack of  understanding of the care planning process and a need for support in understanding how it would proceed. Carers reported needing a clear introduction to the care planning system, including details on how it works in practice, in order for them to be fully involved in the process.

*“when a person becomes a carer, it’s not really explained to them exactly what’s gonna happen.”* Carer 1011

*“…people need to be informed that this is a care plan, this is how we develop it, and this is how it works, this is how it’s implemented. And the problem is that we’re just told that the social worker will develop the care plan.”* Carer 1012

If carers had become involved in the care planning process, there were structural barriers to participation, such as not being invited to attend meetings or only being told about meetings once they had happened. Participants felt that improving access to such meetings and actively inviting carers would facilitate involvement in the meetings.

*“sometimes, I wasn’t told about the reviews until they’d actually happened… and that was really annoying, because there’s things that obviously I’d seen in my son, having visited him every day, and I… could speak… from more knowledge than… the professionals could.”* Carer 1002

However, participants did describe how flexibility of access would also need to be incorporated into the care planning process, to enable involvement from a wider range of carers. For example, one carer described how they had been included in the process despite not being able to attend meetings and that this had been useful.

*“…the social worker came out to see me and explained everything and who’s involved in his care… And wanted my input, even though I couldn’t get down there because of the travelling.”* Carer 1008

One of the key factors that appeared to influence involvement in the process of care planning, was the relationship between carers and health professionals.

*“So, everything depends on the relationship… I don’t think it’s the only thing by any means.. But it’s crucial”.* Carer 1005

The consensus amongst participants was that a good, trusting relationship fostered over time with health professionals could facilitate open information exchange, and make for a positive experience for all parties. This relationship was facilitated by a perceived continuity of care with the health professionals involved in their care.

*“He’s had the, the same care manager and the same support worker for quite a long time, in fact, in fact his support worker has been there from the beginning, he’s more like a family friend really than anything else.”* Carer 1008

However, this type of relationship was not always apparent in the experience of the participants.

### Relational barriers to involvement in care planning

Improving the quality of the care planning culture and process was frequently cited by participants as a way of improving carer involvement in mental health services. Participants talked about a tendency for a power imbalance to exist within consultations between professional and carers. Professionals were seen as the ‘experts’, a view exacerbated by their use of medical terminology, which was considered to widen the perceived divide. Carers often felt that this imbalance was more pronounced in relationships with more senior clinicians.

*“And I think there’s an awful lot of… us and them, and a bit kind of pat you on the head, you’re not expected to know what all this jargon means.”* Carer 1013

*“I think the difficulty will arise with the more senior clinician… because in my experience more senior clinicians can have a very different attitude, and I think that there’s a genuine attitude which says, I’ve studied and trained for a long time to develop this expertise, I know what I’m talking about”.* Carer 1005

This relational aspect was further highlighted by the descriptions of paternalism within the narratives provided by carers, and most participants still perceiving they required permission to participate in decisions about care planning from the health professional. In extreme cases, carers felt that professionals demonstrated an inherent disregard for their views, which could be very distressing for the carer concerned.

*“Now [CONSULTANTNAME] at [HOSPITAL] is a very stern man, fair man, a good consultant, but most people will tell you who've had dealings with him he's very stern, you only speak when he asks you to speak and usually right at the end”.* Carer 1007

### Confidentiality as a barrier to involving carers in mental health care planning

Confidentiality was frequently raised as a barrier to carers becoming involved in both care planning and service users’ care more generally. This was an emotive subject for many carers, and it could often reinforce self- blame for contributing to a service user’s suffering.

*“I think there's still issues around that of… are parents, for example, to blame for the mental illness? Are they a hindrance to the person getting better? There's all sorts of things like that…they're not far under the surface, those kind of things. And certainly when I talk to carers… whose children have been admitted and have chronic, you know, severe episodes and so on… they feel just immediately excluded like, like it's nothing to do with them or… almost made to feel they're the enemy.”* Carer 1014

Some participants described experiences where they perceived that the idea of confidentiality had been misused previously to exclude carers from the service user’s care, which was upsetting for those concerned and reduced the perceived importance of carers within health services. This was often attributed to a lack of understanding of the nuances of confidentiality in practice, rather than a deliberate misuse.

*“So it was like huge barricades up around this trivial information, trivial stuff. So… that in itself as you can imagine, was intensely upsetting and, and infuriating. But it's more that it symbolises this idea that as the carers you're nobody.”* Carer 1015*.*

Despite these strong feelings, there was an acknowledgement that service users did have a right to confidentiality. However, several carers felt that even if the service user did not consent to their involvement they should remain informed about the service user’s care and be involved in a way that did not breach confidentiality. The reason for this related back to the aforementioned point about the valuable and unique contribution that carers could make to a service user’s care.

*“I couldn’t get people to tell me…because of confidentiality, you see, and confidentiality is right. I want confidentiality for my son, I want respect and I want people to listen to him, but also I need to be listened to.”* Carer 1016

Carers spoke of the tensions that could emerge when a service user was acutely unwell and could become hostile towards the carer if they had been involved in the care planning. This became increasingly pertinent if compulsory measures had been used, particularly if these measures had been initiated by the carer, demonstrating the often competing challenges that are inherent in a carer’s role.

*“I think at the point of crisis with my brother, if I was involved in his care plan, he’d have gone ballistic because I was the one that kind of in his eyes got him sectioned and so on”.* Focus group A

Confidentiality was seen as a two-way process and carers sometimes wanted their own discussions around care to be kept confidential. Sometimes this had been respected by professionals, but a small number of carers reported how difficult situations had arisen which had impacted negatively on the service user/carer relationship, when they felt health professionals had breached their own confidentiality, by telling service users things that they had specifically asked them not to. These different standards or foci of confidentiality for professionals reflect the relegation of the carer’s role and prioritisation of the service user within services.

*“… there have been times when, you know, I’ve said things about my son because I felt he was becoming unwell, but I didn’t want the nurse to say to my son … that I’d told him. So… the nurse went and told my son and that made my son very cross and so we had to build bridges again.”* Carer 1001

*“I said I wanted it confidential and she should have listened to me and respected that, because that is the law, I should have my confidentiality, she didn’t keep that and she told my son which made the situation far worse.”* Carer 1016

There was acknowledgement that this is a difficult, complicated area but there were strong feelings that with the appropriate organisational support, carers should have a voice and be able to contribute meaningfully and appropriately to care planning decisions. One suggestion for this support was that in times of conflict between service users and carers, an independent and qualified mediator could make assessments about carer involvement and advise accordingly.

*“I think it’s a really difficult area… that understandably there’s… levels of confidentiality and so on. But I also think the carers… should have a voice. I think it should be assessed by somebody who’s really qualified to assess it and, and weigh the balance up of what the carer’s saying, what the service user needs and is saying, and make a judgement that way. But often carers are not listened to at all and are completely left out of the equation”.* Focus group A

Participants felt that one way to ameliorate the situation would be for professionals to engage carers in an open and honest dialogue, in which they explained in a meaningful way what confidentiality actually meant in their individual situation, but that there should be a consistent approach from all professionals and this would require relevant and up-to-date training.

*“I’m really concerned that people should have training on that, you know confidentiality, that everybody knows what they’re saying, because if one person says one thing, another person says another, then the poor, well the carer in particular is… on a hiding to nothing.* Carer 1008

## Discussion

This paper reports on a qualitative analysis of interviews and focus groups with carers of people with severe mental illness. Compared to service user views, the reasons underpinning carers’ dissatisfaction with care-planning procedures have been relatively neglected in the research literature, despite the substantial and significant contribution that they make to mental health services [33–35]. This is despite the potential contribution they could make across both voluntary and statutory settings [7] and the formal recognition of carers in recent legislation [9, 10, 11, 12–15]. Our data have confirmed that many carers perceive a lack of involvement in care planning and a lack of recognition and appreciation of their role from health professionals. Whilst this finding corroborates national survey data, the emergence of a novel and shared model of optimal carer involvement and the qualitative identification of temporally relevant facilitators and barriers to its instigation are fundamentally important to note.

Historically, research has demonstrated high levels of dissatisfaction among carers regarding their involvement in mental health services [29], including consistent requests for greater information provision and exchange [30]. Our results support and extend these findings, with participant narratives reflecting a shared perception of a statutory service ethos in which carer contributions are devalued and service users are sequestered from their contextual or temporal home environments. Carers describe a hierarchy of care planning influence, in which the relative contributions of different stakeholders are determined more by role status than by their potential expertise. Carer discourse suggests that, whilst not contentious in their own right, such hierarchical approaches risk ambiguity, misinterpretation or misuse.

A subtle but key finding emerging from our work is that carer dissatisfaction is rarely fuelled by an outright rejection of professional accountability for care planning decisions alone. Rather, it stems from a sense of failure among staff to negotiate professional and theoretical concepts of user confidentiality, in order to facilitate holistic, user and carer-centred care [37]. This supports data from the professional arena which demonstrates that professionals are not always clear about the limits of confidentiality and that training relating to specific legal requirements may be of value [38]. The data collected herein extends our understanding of carers’ perceptions of mental health care planning, by demonstrating that, irrespective of their prior experiences, many carers continue to acknowledge the potential value of care plans, both as an aid to service user recovery and a much needed facilitator for stakeholder communication, as well as a statement of the services they can expect service users to receive.

Pertinently, none of the carers participating in our study were seeking to dominate decision-making processes, but felt they had valuable contributions to make to the traditional service user and professional dyad. Specifically, this related to a perceived contextual and holistic understanding of the service user. Neither did they wish to contend service users’ own requests for confidentiality. Rather, they conceived their involvement as an integral element in a flexible and responsive model of mental health care. Particularly evident in this regard were carers’ desires to enhance and contribute to a holistic view of a service user (e.g. physical health), to feel validated in reporting early signs of relapse to mental health professionals, and to be able to function more effectively themselves in their own informal caring roles. The realisation of these foci is likely to demand the development and implementation of dedicated support frameworks capable of managing the variable, fluctuating and sometimes conflicting needs of a stakeholder triad.

Carers in our study felt quite strongly that their successful involvement in care planning could enable and enhance four distinct areas of health service provision: service efficiency, service user understanding, professional communication and direct service user support. This supports research demonstrating the positive impact of involving carers in treatment programmes [21, 22]. It would seem that given this, there is potential value in substitution or advocacy roles being attributed to carers in situations where service users are less able, or willing, to be involved in care planning decisions themselves, with permission obtained from service users (e.g. advancement statements).

Notably, by default of their own position, all carers in the current study tended to view their contributions as expedient. It should be noted that this paper does not report the views of other stakeholders, and as such cannot verify that all carer contributions will be perceived as uniformly positive. Good carer involvement is facilitated by good relationships with staff, effective communication, partnership working and allowing sufficient time for explanations to be given and understood. Yet barriers to involvement are also wide - ranging, and can include high workloads within services, unhelpful staff attitudes, and periods of more severe illness. Participants specifically recognised the inter-personal tensions that could arise when a service user was unwell, and the potential hostility that could be directed towards carers, particularly in instances where these individuals were perceived by service users to have facilitated or condoned the need for their compulsory detainment or treatment. This observation confirms the need to develop and consolidate explicit and transparent communication protocols at an organisational level, perhaps under the guidance of a nominated lead for carer involvement, in order to facilitate and evaluate the impacts of any such initiatives more fully.

Individual interpretations and professional perceptions of patient confidentiality were consistently cited as a major barrier to optimal carer involvement in the care planning process, as has been found previously [37]. The potential for abuse and misuse of this construct was an emotive subject for the participants in our study, often as a result of prior and direct experience. This finding identifies an urgent and joint training need for mental health service users, carers and professionals; the development of consensual understanding being a mandatory step in improving relationships, ensuring consistency and enhancing stakeholder experiences of care planning processes. Successfully embedding any such enterprise into contemporary health services will demand a concurrent shift in organisational ethos, such that the implementation of confidentiality frameworks and procedures is seen as a progressive and valid initiative to lessen reliance on individual competences and attitudes.

## Conclusions

The qualitative study presented here has developed the understanding of the potential role of carers within the care planning process within mental health services, along with the facilitators and barriers to achieving optimal involvement. Given the impetus in current UK policy, and initiatives to improve service user and carer involvement in care planning, it is likely that these findings have direct policy and practice relevance in ensuring that such initiatives realise their full potential and do not culminate into rhetoric.

### Strengths and limitations of the current study

The current study finds its strength in the combination of qualitative research methods undertaken with a wide range of carers. By adopting this approach, we allowed issues raised in focus groups to be explored in more depth in one-to-one interviews. Additionally, the combination of approaches allowed for on the one hand discussion on a groups basis which may derive findings that would not have arisen from one-to-one interviews alone whilst concomitantly engaging carers on a one-to-one basis to ensure issues that participants may not feel able to discuss in a group setting could also be explored. The research team comprised of service users and carers trained in qualitative methods and researchers. The lead author is a carer researcher. This successful collaboration has enabled the paper to provide a unique contribution to existing literature through a contextual understanding of the themes arising from the data. The work builds and extends previous research in this area by providing a framework to recommend practical changes to mental health services.

It should be acknowledged that only the views of carers are presented in the current paper, and it has not been possible to directly compare and contrast findings with data from service users and professionals. Various strategies were incorporated into the study design to enhance the validity of the findings, and the presentation of quotes with themes emerging from the data should enhance the transferability of the findings. However, it should be noted that the themes presented in this paper are only partial views, and the findings therefore remain exploratory in nature. The authors feel that this is justified by the demonstration that current data providing in-depth analysis of carers’ views in their own right is limited, and where it is available, is underrepresented in combined analysis. The views of service users, professionals and other stakeholders have been presented elsewhere.

Given the combined use of qualitative methods, this paper builds on and expands on current knowledge about the experience of carers of people with severe mental illness in mental health services, and care planning in particular.

## Abbreviations

BME: Black and Minority Ethnic
EQUIP: Enhancing the quality of user involved of care planning in Mental Health services
NIHR: National Institute of Health Research
NRES: National Research Ethics Committee
UK: United Kingdom
